# Scalable Nanomanufacturing—A Review

**DOI:** 10.3390/mi8010020

**Published:** 2017-01-11

**Authors:** Khershed Cooper

**Affiliations:** Nanomanufacturing Program, Civil, Mechanical, and Manufacturing Innovation Division, National Science Foundation, Arlington, VA 22230, USA; khcooper@nsf.gov; Tel.: +1-703-292-7017

**Keywords:** scalable nanomanufacturing, scalable nanopatterning, nanoprinting, nanoimprinting, self-assembly, directed assembly

## Abstract

This article describes the field of scalable nanomanufacturing, its importance and need, its research activities and achievements. The National Science Foundation is taking a leading role in fostering basic research in scalable nanomanufacturing (SNM). From this effort several novel nanomanufacturing approaches have been proposed, studied and demonstrated, including scalable nanopatterning. This paper will discuss SNM research areas in materials, processes and applications, scale-up methods with project examples, and manufacturing challenges that need to be addressed to move nanotechnology discoveries closer to the marketplace.

## 1. Introduction

Nanomanufacturing is the manufacture of nano-scale materials, structures, components, devices and systems [1]. It is the manipulation and control of matter at the nano-scale [2,3]. It is the design and fabrication of nanostructures that exploit the unique physical and chemical phenomena that occur at the nano-scale such as quantum and surface effects. Nominally, the dimensional scale for nanomanufacturing is 1–100 nm. However, it is usually a scale, in the sub-micron range, where unusual or enhanced materials behavior occurs that can be exploited in developing a product. Scalable nanomanufacturing is the large-scale manufacture of nanomaterials and nanostructures, their assembly into components, devices and sub-systems, and the integration of these assemblies into higher-order systems. Nanomanufacturing research is the conception and development of physical processes and methods to overcome the scientific and technical barriers that prevent the translation of lab-scale synthesis of nano-scale materials and structures to industrial-scale production. Research in scalable nanomanufacturing addresses manufacturability challenges such as scalability, reliability, controllability, efficiency, quality, yield and affordability. The Scalable Nanomanufacturing (SNM) program at the National Science Foundation (NSF) strives to meet these challenges by seeking research ideas that have the potential to achieve nanomanufacturing scale-up through large-area, continuous and other high-throughput processes. Among the processes investigated is scalable nanopatterning for which methods such as top-down nanoimprinting and bottom-up self-assembly, among others, are exceptionally suitable.

## 2. National Science Foundation (NSF) Scalable Nanomanufacturing

The NSF Scalable Nanomanufacturing (SNM) Solicitation [4] was first announced in 2011 in response to the National Nanotechnology Initiative (NNI) Signature Initiative (NSI) in Sustainable Nanomanufacturing: Creating Industries of the Future [5]. The Sustainable Nanomanufacturing NSI was in response to the recommendation in 2010 of the President’s Council of Advisors on Science and Technology (PCAST) to double research and development investments in nanomanufacturing in five years, the goal being to accelerate commercialization of nanotechnology-enabled products. The goal of the Sustainable Nanomanufacturing NSI is to establish manufacturing technologies for economical and sustainable integration of nano-scale building-blocks into complex, large-scale systems. The objective of SNM is to meet this goal through basic research in novel nano-scale processes towards the fabrication of nanomaterials and nanostructures in large quantities, their integration into nano-enabled systems and, ultimately, the insertion of these systems into products useful to society and the economy. The key requirements of the SNM solicitation are synonymous with those of the Sustainable Nanomanufacturing NSI, which is that the proposed processes have the potential to be scalable to the required throughput and yield, controllable to the required precision and accuracy, sustainable as demonstrated by life-cycle analysis, and safe during production.

The NSF SNM Solicitation fosters research to overcome the key scientific and engineering barriers that prevent the production of useful nanomaterials, nanostructures, devices and systems at an industrially relevant scale, reliably, and at low cost and within environmental, health and safety guidelines. It emphasizes scale-up, high-throughput approaches such as large-area, parallel, continuous roll-to-roll, continuous reactor and other scalable processes. It encourages multi-disciplinary efforts involving disciplines from both engineering and the physical sciences. It encourages tangible industry participation. The focus is on novel scalable processes and methods and fundamental scientific research in well-defined technical areas that are strongly justified as approaches to overcome critical barriers to scale-up and integration of nano-scale processes. Outcomes could be design principles for production systems and enabling tools leading to nanomanufacturing platforms; identification of metrology, instrumentation and standards; development of process monitoring and control methodologies; and creation of product quality and yield assessment tools. SNM seeks to address critical portions of the nanomanufacturing value chain of nano-scale building-block production → nanostructure fabrication → nanocomponent/nanodevice manufacture → nano-subsystem design and manufacture → nanosystem design and integration → product insertion. The overarching goal of the SNM solicitation is to establish the fundamental principles for volume manufacturing of useful nanotechnology-enabled products at low cost.

## 3. SNM Research Areas

The NSF SNM Solicitation has completed 6 years of sustained support of fundamental research in scalable nanomanufacturing. Nearly 50 projects in various research areas have been awarded between 2011 and 2016. All awards along with research abstracts are listed at the end of the solicitation web-page [4]. SNM covers many research areas in nano-scale materials, processes and potential nano-enabled applications, examples of which are given below.

### 3.1. Nano-Scale Materials

- Zero-D: Quantum Dots, Core-shell/Composite/Magnetic Nanoparticles.
- One-D: Nanowires, Carbon Nanotubes, Carbon Nanofibers, Cellulosic Nanocrystals.
- Two-D: Graphene, Transition Metal Dichalcogenides (TMDs), Bucky Tape.
- Three-D: Nanoporous Membranes, Aerogels, Nanostructured Materials.

### 3.2. Nano-Scale Processes

- Chemical/Thermal: Combustion, Plasma, and Hydrothermal Synthesis, Chemical Etching, Thermal Drawing, Microreactor.
- Vapor-based: Chemical Vapor Deposition (CVD), Physical Vapor Deposition (PVD), Plasma Enhanced Chemical Vapor Deposition (PECVD), Atomic Layer Deposition (ALD), Molecular Layer Deposition (MLD).
- Solution-based: Wet and Slot Coating, Film and Laminate Casting, Colloids, Microfluidics, Ink-Jet Printing.
- Electrolytic: Electrospray, Electrophoresis, Electrospinning, Electroetching.
- Lithography/Deposition: Atomic Force Microscope (AFM), Nano Imprint Lithography (NIL), Laser Beam, Electron Beam Lithography (EBL), Ion-Beam, Direct Write.
- Assembly: Self-assembly, Directed-Assembly, Block Copolymer Self-Assembly.
- Bio Nanofabrication: DNA Templating.
- Mechanical: Exfoliation.
- 3D Nanofabrication: 3D Printing, Stereo-Lithography, Strain Engineering.

### 3.3. Potential Nano-Enabled Applications

- Environmental: Water Purification, Analytical Separation, Wastewater Treatment.
- Chemical: Catalysis, Gas Storage.
- Energy: Storage, Conversion, Harvesting, Batteries, Supercapacitors, Photovoltaics (PVs), Solar Cells.
- Electronics: Integrated Circuits (ICs), Flexible, Storage Memory, 3D Devices, Thin-Film Transistors (TFTs), Electromagnetic (EM) Shielding.
- Optoelectronics/Photonics: Imaging, Waveguides, Displays, Lighting, Metamaterials.
- Sensors: Biological, Chemical, Multiplexed.
- Structural: Nanocomposites, High-Strength, Light-Weighting, Packaging.
- Biomedical: Implants, Tissue Scaffolds, Diagnostics, Therapeutics, Probes.
- Sheets/Wires: Fibers, Cables, Filters, Membranes, Textiles, Paper, Fabric, Nonwovens.
- Templates: Masks, Photoresists.

It is clear that nanomanufacturing advances will impact applications across all industrial sectors. The impact can be in the form of new products, improved products, and products with new functionalities. From a materials perspective, several nano-scale building-blocks, each with unique properties and behavior, are available to form nano-scale structures and systems in a variety of forms with a variety of functions. These are the “raw materials” to be manipulated and controlled by nanomanufacturing processes to form nanostructures and nanosystems. A recent inventory [6] of nanomanufacturing processes counted in excess of 150 processes, many falling under the bottom-up (assembly) paradigm and many more falling under the top-down (lithography) paradigm. The take-away from the above analysis is that a wide range of nano-scale materials and processes are available to build nano-scale structures and integrate them into large-scale systems. The basic research efforts will be in selecting an appropriate mix of these materials and processes that would eventually result in useful products.

## 4. Scaling-Up

Scale-up of nanomanufacturing processes is achieved through one or a combination of several approaches.

(1) Continuous Roll-to-Roll Top-down/Bottom-up Processes: printing, imprinting, self-assembly, deposition, coating, lamination.
(2) Parallel, Large-area Top-down/Bottom-up Processes: lithography, direct-write, directed- and self-assembly.
(3) Parallel, Large-area 3D Nanofabrication: nano 3D printing, 2-photon polymerization, nanoimprinting and self-assembly, strain engineering.
(4) Large-area DNA Nanofabrication: templating using DNA.
(5) Semi-continuous, Continuous or Parallel Chemical/Fluid/Thermal Techniques: microreactor, microfluidic, electrospray, electrospinning, fiber-drawing.

## 5. Scalable Nanopatterning

By definition, nanopatterning is the fabrication of 2D and 3D nanostructures with recurring or repeated design. Nanopatterns can be created in different sizes and shapes. They can be uniform, gradient or variable. Nanopatterning can be done using top-down or bottom-up processes or a combination of the two. Both 2D (surface) and 3D (volume) nanopatterning have been demonstrated using a variety of processes. Scalable nanopatterning involves addressing manufacturability challenges such as throughput, scalability, regularity, controllability, quality and yield. While electronic and optoelectronic applications may be the primary beneficiaries of successful research in scalable nanopatterning, non-electronic applications would also benefit. The goal is to come up with manufacturing platforms for scalable nanopatterning that are versatile and reconfigurable and that could be used to make different patterns of different materials for a variety of applications. Of the five general approaches listed above, the first four are suitable for scalable nanopatterning.

### Scalable Nanopatterning Research at NSF

Scalable nanopatterning approaches are exemplified by many ongoing NSF SNM research projects, a list of which can be found at the end of the SNM Solicitation description [4]. The list contains the project title, investigator names, the primary institute and an abstract which briefly describes the project.

Examples of SNM research projects addressing scalable nanopatterning challenges are many. The projects fall under one of four general scalable nanomanufacturing technologies defined above as follows:

(1) Continuous Roll-to-Roll (R2R) Top-Down/Bottom-Up Processes.

Sreenivasan, Bonnecaze and Grant’s group [7,8,9] is developing ink-jet based nanoimprint lithography and block copolymer self-assembly on amorphous silicon to form thin film photovoltaics. Gilchrist’s group [10,11] is studying nanoparticle monolayer self-assembly and convective Langmuir-Blodgett film deposition methods to fabricate nanoporous membranes, flexible dye sensitized solar cells, and light-emitting diodes. Guo and Hart’s group [12,13] is studying continuous CVD synthesis and patterning of CNT and graphene on flexible substrates for electronic and optoelectronic applications. Osuji’s group [14,15] is investigating large-area processing with controlled nanoporosity of aligned CNT and nanoporous membranes for analytical separation and water purification. Lee’s group [16] is developing continuous atomic and molecular layer deposition techniques with process control of conformal and ultrathin inorganic and organic films for gas diffusion-barrier and Li-ion battery electrode coatings. Zorman’s group [17] is developing a continuous microplasma-based direct write fabrication of sub-100 nm patterns of metallic and metal oxide structures for functional devices on flexible substrates. Raman and Fisher’s group [18] is investigating continuous high-energy PECVD growth of functionalized graphene nanopetals for applications such as energy storage, biosensor and nano-composites. Chang’s group [19] is developing a continuous system that includes microreactor-assisted semiconductor nanoparticle synthesis, ink formulation and deposition and flash-light sintering to pattern thin films for devices on large area flexible substrates. Lee’s group [20,21] is studying continuous slot coating of particle suspensions and nanoimprint lithography of thin-film composite nanostructured membranes for wastewater treatment and water purification. You’s group [22,23] is developing low-temperature, high-throughput wet coating processes to produce polymer solar cells/nanowire electrodes and supercapacitors for energy conversion and storage devices. 

(2) Parallel, Large-Area Top-Down/Bottom-Up Processes. 

Fang’s group [24,25] is developing digital in-flow nanoimprint lithography of heterogeneous metablocks and directed self-assembly by plasmonic enhanced parallel optical trapping of heterogeneous metamaterials for tele-communication, quantum computing and energy applications. Xu’s group [26] is developing parallel nanoimprint lithography and nanomaterials synthesis using nanoscale optical antennae combined with in-line metrology and system integration of multi-scale nanodevices for chem/bio sensing and next-generation data storage. Nealey’s group [27] is investigating CVD and directed self-assembly of block copolymer on lithographic chemical pre-patterns for 5 nm resolution nanoimprint lithography to fabricate faster computer chips and high storage bit-patterned media. Alexander-Katz’s group [28] is studying block copolymer self-assembly on graphoepitaxial templates of heterogeneous hierarchical nanomaterials to create optimal templates for targeted patterns. Fourkas’ group [29] is developing three-color photolithography of high resolution nanoscale photoresists for high density integrated circuits for 2D and 3D semiconductor devices. Ragan and Boyraz’s group [30] is investigating directed self-assembly of colloidal nanoparticles and electrospun direct write of nanowires for nanoarchitectures with sub-λ metallic building blocks for functional devices for sensing, imaging and light-guiding. Xu’s group [31] is developing anodized aluminum coloration by surface nanostructuring and nanoparticle infiltration of nanopores for light and color manipulation for automotive, aerospace, and consumer product applications. Anthamatten and Shestopalov’s group [32,33] is developing additive contact printing with shape memory polymer stamp having responsive topography of patterned organic and inorganic thin films and their multilayered stacks for clean, defect-free and high resolution Organic Light-Emitting Device (OLED) arrays. Sun’s group [34] is studying hierarchical block copolymer assembly of nanocomposite materials and light-trapping coatings for thin film solar cells and smart-window coatings. 

(3) Parallel, Large-Area 3D Nanofabrication. 

Chen’s group [35,36,37] is investigating hyperlens-assisted projection stereo-lithography and direct write of 3D heterogeneous biological scaffolds using biodegradable materials for tissue engineering applications. Grigoropoulos’ group [38] is investigating directed self-assembly of block copolymers and laser processing for multi-scale scaffold structures of complex 3D metamaterials for optical waveguides and high sensitivity sensors. 

(4) Parallel, Large-Area DNA Nanofabrication. 

Slinker’s group [39] is investigating molecular building-block assembly on DNA-like templates and bread-boarding with nanoimprint lithography of organic semiconductor nanowires for nanoscale electronic circuit elements. Hughes’ group [40,41] is developing molecular self-assembly of atomically-precise, defect-free DNA patterns with in-line optical metrology of engineered masks for semiconductors and memory devices. 

## 6. Manufacturing Challenges

Manufacturing challenges fall in two categories. Desired outcomes and appropriate metrics. The desired outcomes are product quality and durability, process repeatability and reliability, production scalability and affordability, production efficiency and yield, and product performance and functionality. Precision of placement, feature size and resolution, overlay registration and nanostructure density, complexity and their rates of forming are some of the metrics that need to be determined. These are challenges for all manufacturing processes, but more so for nanomanufacturing because manipulating, measuring and controlling at the nano-scale is not easy and small errors can result in large failures. In addition, a compromise needs to be made when considering feature size and resolution in the context of processing or forming rates and high volume production. 

Furthermore, each nano-scale process is unique, generally untested and needs validation. Each nanocomponent may have a unique processing history and needs validation. To attract commercial interest, extensive proven history, reliable supply chain, universal standards and targeted metrics are needed. Toxicity, environmental, health and safety standards and regulations need to be in place. Finally, the question: “Is there a market for the nano-enabled product?” needs to be asked and answered.

## 7. Discussion

Scalable nanomanufacturing and its sub-set scalable nanopatterning are the bed-rock technologies for volume manufacturing of structures and components for nano-enabled products. Research in these areas can lead to the development of new, nanomanufacturing platforms that are capable of making a variety of structures and patterns for integration into devices and systems. The NSF SNM solicitation’s goal is to create the fundamental principles for scalable nanomanufacturing, which would accelerate the translation of lab-scale discoveries and inventions to the marketplace. Scale-up approaches involve processes such as continuous roll-to-roll top-down/bottom-up, parallel large-area top-down/bottom-up, parallel large-area 3D nanofabrication and large-area DNA nanofabrication. As the above research examples show methods are being developed that involve a variety of material sets and nanomanufacturing processes for applications impacting all technological sectors.

## 8. Conclusions

The NSF Scalable Nanomanufacturing (SNM) Solicitation’s objective is to investigate a wide range of key research areas in nanotechnology and nanomanufacturing from fundamentals to real world issues and impacts. Its focus is on basic research that is high-risk and related to *industrially relevant scale nanomanufacturing* that can *enable high-value end applications*. There are persistent knowledge gaps that must be resolved to translate the prospects of emerging nanotechnologies and nanomaterial-enabled applications into practice, which SNM tries to close. Scalable nanopatterning is a sub-set of SNM and is well represented in NSF supported SNM projects. Scalability in nanopatterning is achieved by one of several means involving either continuous roll-to-roll or parallel, large-area processes such as printing and imprinting, lithography and self-assembly, and 3D and DNA nanofabrication. SNM research projects are developing fundamental principles for design rules, materials selection, processing routes, and measurement and control methodologies based on models and simulations and assessments of quality and yield. These fundamental principles should lead to nanomanufacturing platforms for several technologies including scalable nanopatterning.
